# Automatic Recognition of Ripening Tomatoes by Combining Multi-Feature Fusion with a Bi-Layer Classification Strategy for Harvesting Robots

**DOI:** 10.3390/s19030612

**Published:** 2019-02-01

**Authors:** Jingui Wu, Baohua Zhang, Jun Zhou, Yingjun Xiong, Baoxing Gu, Xiaolong Yang

**Affiliations:** 1 College of Engineering, Nanjing Agricultural University, Nanjing 210031, China; 15895928166@163.com (J.W.); zhoujunnjau@126.com (J.Z.); xyj@njau.edu.cn (Y.X.); gbx@njau.edu.cn (B.G.); 2 College of Horticulture, Shenyang Agricultural University, Shenyang 110866, China; xiaolongyang0214@163.com

**Keywords:** tomato recognition, harvesting robots, multi-feature fusion, feature analysis, weighted RVM classifier, I-RELIEF, two-layer classification strategy

## Abstract

Automatic recognition of ripening tomatoes is a main hurdle precluding the replacement of manual labour by robotic harvesting. In this paper, we present a novel automatic algorithm for recognition of ripening tomatoes using an improved method that combines multiple features, feature analysis and selection, a weighted relevance vector machine (RVM) classifier, and a bi-layer classification strategy. The algorithm operates using a two-layer strategy. The first-layer classification strategy aims to identify tomato-containing regions in images using the colour difference information. The second classification strategy is based on a classifier that is trained on multi-medium features. In our proposed algorithm, to simplify the calculation and to improve the recognition efficiency, the processed images are divided into 9 × 9 pixel blocks, and these blocks, rather than single pixels, are considered as the basic units in the classification task. Six colour-related features, namely the Red (R), Green (G), Blue (B), Hue (H), Saturation (S) and Intensity (I) components, respectively, colour components, and five textural features (entropy, energy, correlation, inertial moment and local smoothing) were extracted from pixel blocks. Relevant features and their weights were analysed using the iterative RELIEF (I-RELIEF) algorithm. The image blocks were classified into different categories using a weighted RVM classifier based on the selected relevant features. The final results of tomato recognition were determined by combining the block classification results and the bi-layer classification strategy. The algorithm demonstrated the detection accuracy of 94.90% on 120 images, this suggests that the proposed algorithm is effective and suitable for tomato detection

## 1. Introduction

Tomato is a popularly cultivated fruit/vegetable that is highly favoured by consumers worldwide owing to its unique flavour, rich nutritional content, and health-promoting properties. Automation technology has been widely used in various fields, such as machinery manufacture, industrial production, traffic control, and agriculture. The rationale for agricultural automatization is reduction in the manpower; consequently, robotized harvesting has become popular in agriculture automatization [1]. In China, harvesting of tomatoes is associated with high labour cost. At the same time, tardy manual work causes deterioration, and improper handling during plucking may influence the transportation and preservation of tomatoes. There has been a recent trend of replacing human workers with harvesting robots to avoid the above-described drawbacks [2]. For autonomous harvesting, recognition and localization of fruits and vegetables is fundamentally important [3]. Therefore, efficient and automatic identification of ripening and ripe tomatoes is essential for achieving automatized harvesting [4].

The vision unit of a robot is essential for identification and localization of objects, and therefore is an important component of harvesting robots [5,6]. With the development of sensor technology, the image acquisition equipment used in studies has also transited from the black-and-white camera originally to the visual sensor currently. Charge Coupled Device (CCD) and Complementary Metal Oxide Semiconductor (CMOS) are the sensors most commonly used as vision units. In addition, structured-light vision system, spectral cameras and thermal cameras have their own advantages in different situations (e.g., [5,7,8]). Moreover, the combination of multi-sensors may achieve a better performance during acquiring images. In this paper, we used the Live MOS sensor (Live MOS is an image sensor with the same functions as CCD and CMOS). This sensor not only has the better imaging effect advantage of CCD, but also the characteristic less power consumption of CMOS. We use this sensor to obtain higher quality experimental samples to pursue better experimental process.

Recently, machine vision and automatic recognition algorithms for harvesting robots have been studied by many scholars, owing to the clear advantages of automatized picking (e.g., [9,10,11,12,13,14,15,16,17,18,19]). The first proposed method utilized basic graphical information. Colour composition is the basis of an image, equally important for identification of all vegetables and fruits; therefore, colour-based models have been most commonly studied (e.g., [20,21,22,23,24,25,26,27,28]). In particular, Xu et al. [22] used a colour-based model to analyse the fruit’s colour information, and red-blue (R-B) chromatic aberration information of images has been used for recognizing oranges hanging from a tree. Nguyen et al. [29,30] used multi-color model and high- dimensional feature space for image segmentation. Arefi et al. [31] combined the differences among the ripe tomato (RT), unripe tomato (UT) and background with erosion separation to complete the identification of ripe tomatoes. Later, morphological analysis was applied to complement colour features by providing shape information for identification of tomatoes in images, because the above-mentioned basic information methods failed to achieve satisfactory recognition on images with certain non-trivial backgrounds [23]. Plebe and Grasso [21] applied the hue saturation value (HSV) colour space method and combined it with adaptive shape analysis for recognizing the images of harvesting robots. Hannan and Bulanon [23] developed a machine vision system that combined a colour model, adaptive segmentation, and shape analysis for detection of green and red oranges. The Hough transform method, based on the idea of shape model, was used to simplify the process of target identification [32]. Texture can also be useful for identification, owing to its importance in visual perception; consequently, many scholars explored that venue (e.g., [27,33,34]). Recently, both the accuracy of fruit recognition and the performance of a detection system were significantly improved using machine learning methods [35]. Application of machine learning methods to the recognition of fruits and vegetables has become very popular (e.g., [25,27,36,37,38,39]). In their review, Gongal et al. [40] summarized machine learning methods for fruit recognition, including unsupervised classification, supervised classification, and soft computing methods, and discussed the advantages and drawbacks of different classifiers. Bulanon et al. [37] used K-means clustering for detection of red apples. However, the classification accuracy was negatively affected by changing background lighting conditions. Meanwhile, Yin et al. [41] segmented the ripe tomatoes by K-means clustering using the L*a*b* colour space. However, the calculation time can be reduced. Chinchuluun et al. [38] eliminated the harmful effects of light condition on detection of citrus fruits, using a supervised classifier. This solution provided a method that used a Bayesian classifier based on the ‘S’ and ‘I’ components of the HSV colour space. However, the above-mentioned methods are not sufficiently accurate for practical applications. Ji et al. [25] used a support vector machine (SVM) to improve the accuracy and efficiency of apples’ recognition. Nguyen et al. [42] propose an enhanced k-NN algorithm used in robust biometric recognition which improve the performance of k-NN classifier obviously. Dubey and Jalal [27] classified fruits and vegetables into one of several classes using a trained multi-class SVM (MSVM), which combined colour and texture to achieve more accurate results for machine learning-based classification of fruits and vegetables. Recently, the application of deep learning has occurred in many studies. Bargoti and Unterwood [16] used multiscale multilayered perceptrons (MLP) and convolutional neural networks (CNN) to realize the image segmentation for fruit detection and yield estimation. Nevertheless, there were segmentation errors in regions with poor image quality due to adverse illumination conditions. The same year, Inkyu et al. [43] achieved the detection of sweet pepper through F-CNN (Fast-Convolutional Neural Networks). It has some obsession of incomplete identification in results. In 2017, Bargoti and Unterwood [44] improved CNN to Faster Regions with Convolutional Neural Network (F-R-CNN) and obtained ideal fruit (including mangoes, almonds and apples) recognition results.

Many attempts have been made to detect fruits and vegetables using computer vision systems, and many related research papers have been published in the past decade; however, many challenges remain. The obstacles mainly include treating complex scenes with variable illumination conditions, foreground occlusions, cluttered backgrounds, random distributions of targets, and irregular cluster-shapes of target objects [5,45]. For ripe tomatoes and their surroundings, one approach that colour and shape analyses was able to detect tomatoes. Wan et al. [18] and Arefi et al. [31] used the colour model and shape analyses to realize the recognition and segmentation of tomato regions with outstanding accuracy. However, colour and shape analyses are not sufficient for avoiding barriers with similar colour characteristics and shapes as tomatoes in our study. From the feature application, individual analysis of colour features is not suitable for our algorithm due to the image interference factors were complex. Therefore, more features need to be extracted to complete our algorithm. Texture features should be used along with colour features to differentiate between tomatoes and other similar backgrounds. Furthermore, if image processing (including feature extraction and pixel classification) is based on the grey values of pixels, computation time required for classification of high-resolution images using conventional sorting techniques is likely to increase. Consequently, the current recognition efficiency is not satisfactory for practical applications. To increase the recognition efficiency, we proposed to use 9 × 9 pixels block as processing unit instead of individual pixel, for algorithm optimization. In addition, machine learning methods are likely to increase the accuracy of image segmentation. On the other hand, different characteristics affect classifiers to different extents. If all characteristics are considered to affect a classifier to the same extent, the classifier would not be able to identify target regions by differentially exploiting the characteristics. The I-RELIEF algorithm was used along with a weighted RVM classifier and relevant features to enhance the recognition efficiency [46].

The goal of the present paper is to develop a method for identification of ripening tomatoes in complex environments, fusing improved multiple features and a bi-layer classification strategy. To achieve this objective, several objectives have to be met: (1) extraction of training features from pixel blocks rather than single pixels, to improve the extraction rate and to reduce the noise pixel interference; (2) processing of unrecognized test images using colour analysis, to process mask images by filtering the colour difference analysis parameters, which is regarded as a first layer classification strategy; (3) evaluating the contribution of extracted features (colour and texture features) using weight analysis based on the I-RELIEF algorithm; (4) training the RVM classifier using features obtained after the weighting process; (5) obtaining the resulting mask images by identification of test images using the weighted RVM classifier, which is regarded as a second layer classification strategy; (6) combining the resulting mask images with processed mask images to obtain the target regions’ mask images; (7) filling the target region mask images based on morphological processing to achieve region identification; (8) marking the target regions in the original test images.

## 2. Materials and Methods

### 2.1. Acquisition of Images

For image acquisition, a Live Metal Oxide Semiconductor (Live MOS; 17.3 × 13.0 mm size) camera (E-P3, Olympus, Tokyo, Japan) was used to take images of tomato fruit. The fruit used for imaging were randomly selected from greenhouse and images of tomatoes were obtained under natural daylight conditions (07:00–16:00) during sunny days. A total of 120 images were obtained manually in December 2017 in the Shenyang Agricultural University. Tomato plants in the greenhouse can reach a height of more than three meters, and the shooting distance concentrated at 30–70 cm due to the narrow space between two adjacent rows. Thus, tomato scenes were randomly selected between the rows of tomato plants. In addition, the shooting will adjust the angle with fruit to adapt more conditions follow the lighting conditions and background of tomato plants. The shooting angle cannot be controlled within a certain range for acquiring more images in different conditions and overcome the problem that too-short distance between the tomato plants at the same time. Identification of tomato is challenged by many external factors (e.g., illumination intensity, overlap between tomatoes or fruits and leaves or stems, backgrounds such as ground, sky, and the constructive framework of greenhouses). Thus, two types of images were obtained, corresponding to different situations: (1) the sunny side and (2) the shadow side. The image resolution was 4032 × 3024 pixels (approximately 12 Megapixels). From the 120 images obtained (there were 60, 60 under the type (1) and type (2) respectively), 30 images were randomly selected for training and the rest of 90 images were used for validation or testing.

The computer which was used to process and analyse the images had an Intel (R) Core (TM) i5-5257U, 2.70 GHz CPU, and was equipped with 8 G of random access memory. The operating system was Microsoft Windows 10, and the software platform was Visual C++ 6.0 and Open Source Computer Vision library (OpenCV).

### 2.2. Algorithm for Automatic Recognition of Ripening Tomatoes

**Figure 1:**
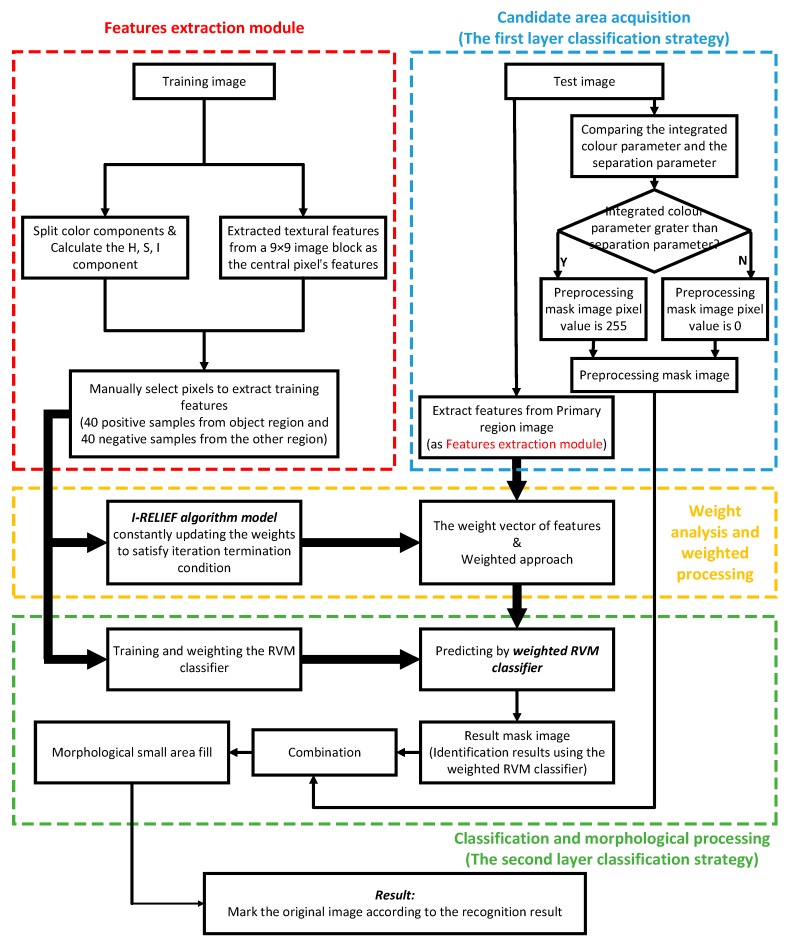
Flowchart of the algorithm for automatic recognition of ripening tomatoes.

In the present work, the proposed image processing and classification algorithm for automatic recognition of ripening tomatoes was developed using a fusion of improved multiple features and a bi-layer classification strategy. The proposed image processing and classification algorithm consists of several parts: (1) image acquisition: For different tomato samples, all images in one type are obtained synchronously and have the same or very similar light intensity; (2) candidate area acquisition: Candidate regions and most other regions have very distinct weights of different components in the colour model. Thus, preselected regions were obtained by combining the R, G, B components in the colour model; (3) feature extraction: Features that were mentioned in Section 2.4. were extracted from training images and test images, respectively. The features from training images constituted the training samples, which were used to train the RVM classifier and to calculate the weight vector. The features from test images constituted the test samples, which were used to determine the classification of test pixels; (4) analysis of weights and weighting: The weight vector was obtained by performing weight analysis on the training samples based on the I-RELIEF algorithm, to improve the RVM classifier training. Meanwhile, the test samples were weighted according to the weight vector, to improve the classification efficiency and accuracy; (5) classification and morphological processing: The RVM classifier was trained using the training samples and the weight vector, following which the classifier was tested using the test set samples. Finally, morphological information was used for optimizing the classification results. The flowchart of the detection algorithm is shown in Figure 1.

### 2.3. Chromatic Difference Analysis

Because images of tomatoes contain a prominent red colour component, the intensity of tomato images is lower than that of their background. Therefore, a fraction of images can be roughly separated to realize the removal of the background by using the red, green, and blue components. During this process, there is a need to ensure that images with the background removed contain complete images of tomatoes as in the original image. We assumed an integrated colour parameter that can be calculated by Equation (1):

(1) $$\begin{array}{ll} \mathrm{The}\;\mathrm{integrated} & \mathrm{colour}\;\mathrm{parameter}\\ & \;=\alpha *R\;\mathrm{component}-\beta *B\;\mathrm{component}-\gamma *G\;\mathrm{component}\; \end{array}$$

where α, β, γ are three different constants that can be adjusted according to the proportion of red, blue, and green components in the image, correspondingly. 

As the threshold condition to separate the target region and the non-target region. Threshold processing is commonly employed in image processing (e.g., [4,47]), and there is a need to ensure that all parts of tomato images are accurately separated. Removal of the background is performed according to the colour parameter of Equation (2):

(2) $$\begin{array}{l} IMG_{mask}\left(X,Y\right)\\ =\{\begin{array}{l} 255,\;integrated\;colour\;parameter\;in\;IMG_{org}\left(X,Y\right)>separation\;parameter\\ 0,\;otherwise \end{array} \end{array}$$

where $IMG_{mask}\left(X,Y\right)$ is the mask image, and $IMG_{org}\left(X,Y\right)$ is the original RGB image. Using this property of tomato images, we can quickly extract approximate tomato images, which expedites the following analysis.

### 2.4. Feature Extraction

The actual target region in an image can be detected using the image’s features. Therefore, feature extraction is an important step in image processing. Except for the RGB colour model, the HIS colour model can fit most lighting conditions and is more suitable for colour perception [25]. Texture-related features can also help to separate the images of fruits from background [40]. Thus, in our research, we extracted six average colour features (including the “R”, “G”, “B”, “H”, “S”, “I’ components extracted from RGB and HIS colour models) and five average textural features (including “Entropy”, “Energy”, “Correlation”, “Inertia-Moment”, “Local-Smooth” features based on the Grey-level Co-occurrence Matrix) from a preselected area used in training.

Among the six colour features (R, G, B, H, S, and I components), R, G, B components can be extracted directly from RGB images and H, S, I components can be calculated through R, G, B components. The function that calculates the H, S, I components is shown in Equation (3):

(3) $$\mathrm{HSI}\;\mathrm{component}\{\begin{array}{l} I\;=\frac{R+B+G}{3}\\ H=\cos^{-1}\left\{\frac{0.5\left[\left(R-G\right)+\left(R-B\right)\right]}{\sqrt{{\left(R-G\right)}^{2}+\left(R-B\right)\left(G-B\right)}}\right\}\\ S\;=1-\frac{3}{R+G+B}\left\{\min\left(R,G,B\right)\right\} \end{array}$$

Texture-related features were extracted from the R component image based on the co-occurrence matrix. To simplify the calculation, textural features, including entropy, energy, correlation, inertia moment, and local smoothing, were extracted from 9 × 9 image blocks, and all of the extracted textural features were considered to be the central pixel’s textural features. Average textural features of a candidate defect region were calculated by averaging the textural features of all of the pixels in that region. The formulas that were used to calculate these textural features are shown in Equation (4):

(4) $$\mathrm{Textural}\;\mathrm{features}\{\begin{array}{l} \mathrm{Entropy}\left(E_{t}\right)=-\sum_{i=1}^{k}\sum_{j=1}^{k}G\left(i,j\right)lgG\left(i,j\right)\\ \mathrm{Energy}\left(E_{n}\right)=\sum_{i=1}^{k}\sum_{j=1}^{k}{\left(G\left(i,j\right)\right)}^{2}\\ \mathrm{Correlation}\left(C_{r}\right)=\sum_{i=1}^{k}\sum_{j=1}^{k}\frac{\left(ij\right)G\left(i,j\right)-u_{i}u_{j}}{S_{i}S_{j}}\\ \mathrm{InertiaMoment}\left(I_{m}\right)=\sum_{i=1}^{k}\sum_{j=1}^{k}{\left(i-j\right)}^{2}G\left(i,j\right)\\ \mathrm{LocalSmooth}\left(L_{s}\right)=\sum_{i=1}^{k}\sum_{j=1}^{k}\frac{G\left(i,j\right)}{1+{\left(i-j\right)}^{2}} \end{array}$$

where:

$$\begin{array}{l} u_{i}=\sum_{i=1}^{k}\sum_{j=1}^{k}iG\left(i,j\right)\;u_{j}=\sum_{i=1}^{k}\sum_{j=1}^{k}jG\left(i,j\right)\;S_{i}^{2}=\sum_{i=1}^{k}\sum_{j=1}^{k}G\left(i,j\right){\left(i-u_{i}\right)}^{2}\\ S_{j}^{2}=\sum_{i=1}^{k}\sum_{j=1}^{k}G\left(i,j\right){\left(j-u_{j}\right)}^{2} \end{array}$$

where G(i,j) is the co-occurrence matrix and k is the dimension of the square co-occurrence matrix. Note that, for calculations, all of the features had to be normalized to the [0,1] interval, to improve the classification performance.

### 2.5. Feature Contribution Ratio Calculation

Determination of the impact of feature contribution ratio on the method’s classification performance was necessary before using the eleven features extracted from images, as mentioned in Section 2.4. In practice, feature-feature correlations are commonly not known in advance, which often leads to situations in which extracted features contain irrelevant features [48]. In other words, for these features, not all features are relevant, and different features contribute differently to the process of classification. Different features contribute to different degrees to the process of classification, with some features contributing more than others. Not only the accuracy of the classification algorithm is affected, but also the calculation process of the classifier becomes redundant if there are irrelevant or insignificant features; this, in turn, reduces the classification efficiency.

Among numerous the judgment method of feature contribution radio, RELIEF is recognized as a simple and effective algorithm. Especially, Sun [49] proposed an iterative RELIEF (I-RELIEF) feature weighting algorithm, solved the problems that the nearest neighbors are defined in the original feature space and the lacking of mechanism to deal with outlier data, which the traditional RELIEF algorithm cannot deal with. In I-RELIEF algorithm, the objective function is optimized through iteration. The iteration will be interrupted until the iteration terminating conditions is met. More detailed formulation of I-RELIEF algorithm presented by Sun [49]. In this paper, we used the I-RELIEF algorithm to determine the contribution ratios of the eleven extracted features (R, G, B, H, S, I, entropy, energy, correlation, inertia-moment, and local smoothing) for selecting the most relevant features among these and for determining the weight coefficients of the RVM classifier.

### 2.6. Related Vector Machine Classifier

Machine classifiers based on feature vectors are the most popular supervised learning models with associated learning algorithms that analyse and recognize patterns used for classification [50]. SVMs and RVMs are commonly used for classification.

Many successful classification applications suggest that SVMs are among the most efficient supervised models for pattern recognition. However, SVM-based methods also have many important and practical shortcomings. First, because the number of support vectors increases linearly with increasing the size of the training set, the basic functions of SVMs are unnecessary in some situations. Second, from both the data and computation perspectives, estimation of the error/margin trade-off parameter in the SVM during cross-validation is not efficient. Third, the kernel function must satisfy Mercer’s condition [51,52].

The RVM has the same function form as the SVM form based on a Bayesian framework. But unlike a SVM, a RVM is a machine learning technique that uses Bayesian inference to provide probabilistic predictions. Therefore, the probability prediction of RVM can judge black or white decision more flexibly according to the actual situation. Wei et al. [53,54] and Demir [55] discussed RVMs in more detail. This is the reason that we chose RVM as our classifier. 

K-NN is a common supervised learning method, and its recognition results and time are worth considering. Its training stage begins after receiving test samples. For the difficulties we encountered in the research, we need to train the classifier using the feature vectors firstly, and then used the testing images as test set for evaluating the proposed algorithm. Therefore, we used the RVM classifier in that it’s more appropriate for our research.

Moreover, different features affect the classification process to different extents; in other words, some features are more relevant for a certain class, while other features are less relevant for that same class. This problem can be dealt with by assigning a real-valued number to capture the degree of relevance of each feature, rather than selecting features using binary values (0 or 1). In the present work, real-valued weights as proportionality coefficients were assigned to each feature, as described in Section 2.5. Using weighted features in RVM classifiers is expected to yield better results.

### 2.7. Novelty and Contributions

**Figure 2:**
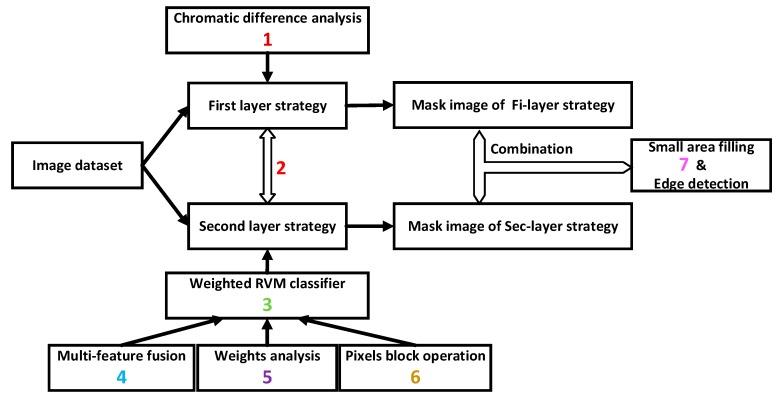
Advantages analysis flowchart of our proposed algorithm.

For the algorithm in this paper, there are several advantages. The analysis about the advance following the sequence numbers in the flowchart (shown in Figure 2).

(1) Compared with the traditional method of using component R individually in image processing, we used chromatic difference analysis as Equation (1) to replace it. The advantage of chromatic difference analysis is that the components R, G, and B are considered in the segmentation process at the same time. The result will be affected observably when using component R individually due to the interference from external factors makes the value of component R change flexibly. The negative influence of external factors will be reduced by subtraction during the chromatic difference analysis.
(2) We separated the operations of the first and the second layer strategies in the study. The second layer strategy won’t deal with the results from the first layer strategy, so as to avoid the error from former strategy affecting the results of later strategy directly.
(3) In the algorithm, we chose RVM classifier because of its kernel function has loosened constraint conditions. In addition, the construction process of RVM classifier is convenient and the effect of identifying is ideal. The application of RVM classifier can also be a peculiarity of this study.
(4) We selected 11 dimensional features which not barely include colour features, but also include textural features in the phase of features selection. The addition of textural features improves the accuracy and applicability of the RVM classifier.
(5) We determined the feature contribution ratio by weight analysis based on the iterative RELIEF feature weighting algorithm. The weighted vector takes the contribution ratio of 11 features into the process of RVM classifier training, which shows the specific character of different features. Neglecting the contribution ratio of 11 features is imprecise and we have already avoided that by this section.
(6) Obviously, the manipulation of using the 9 × 9 pixels black instead of a single pixel used in feature extraction and machine learning classification sections in the study. This substitution can improve the operating speed and computing efficiency at the same time.
(7) In the section of small area filling, we use the boundary points of the region as filter objects. We didn’t choose the method realize the purpose of small area filling by screening the area of connected regions, because of the difficulty of the connecting type and the speed of connected domain judgement is slowly.

## 3. Results and Discussions

### 3.1. Segmentation Based on Colour Difference Analysis 

Using Equations (1) and (2), the first layer classification strategy was successfully realized based on the various colour components from the colour module. In the study, the three constants (α, β, γ) in Equation (1) were set to 1.00, 0.25, and 1.00. The separation parameter in Equation (2) was set to 10. 

We determined the constants and value of threshold by analysing 600 samples pixel-points (200 extracted from tomato regions; 200 extracted from green regions (including leaves and stems); 200 extracted from non-target regions, and we set up the three points sets respectively in groups M, N and K), which is an easy way to find the appropriate values supporting our study.

**Figure 3:**
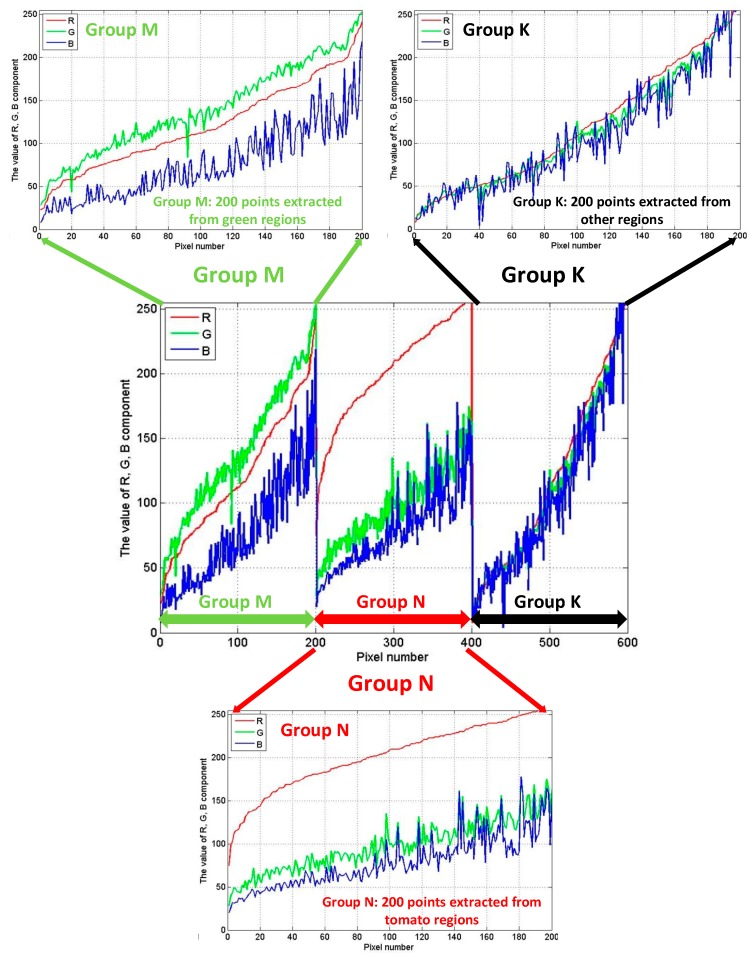
The R, G, B components of three groups M, N and K.

First, we analysed the R, G, B components of three groups (shown in Figure 3). From the Figure 3, we known that the component B runs through the three groups, it has similar influence on the pixel samples with others components in some cases. However, the focus of distinguishing the target regions from the non-target regions is comparison between components R and G. Therefore, using constant parameter *β* to reduce the influence of component B can present the comparison between R and G more clearly. In addition, the influence of component B on the pixel samples is significant, we can reduce the proportion of component B at least, but cannot completely ignore its influence. Hence, we set the parameter *β* to 0.25 which reduced the influence of component B greatly and ensured it’s existent at the same time.

**Figure 4:**
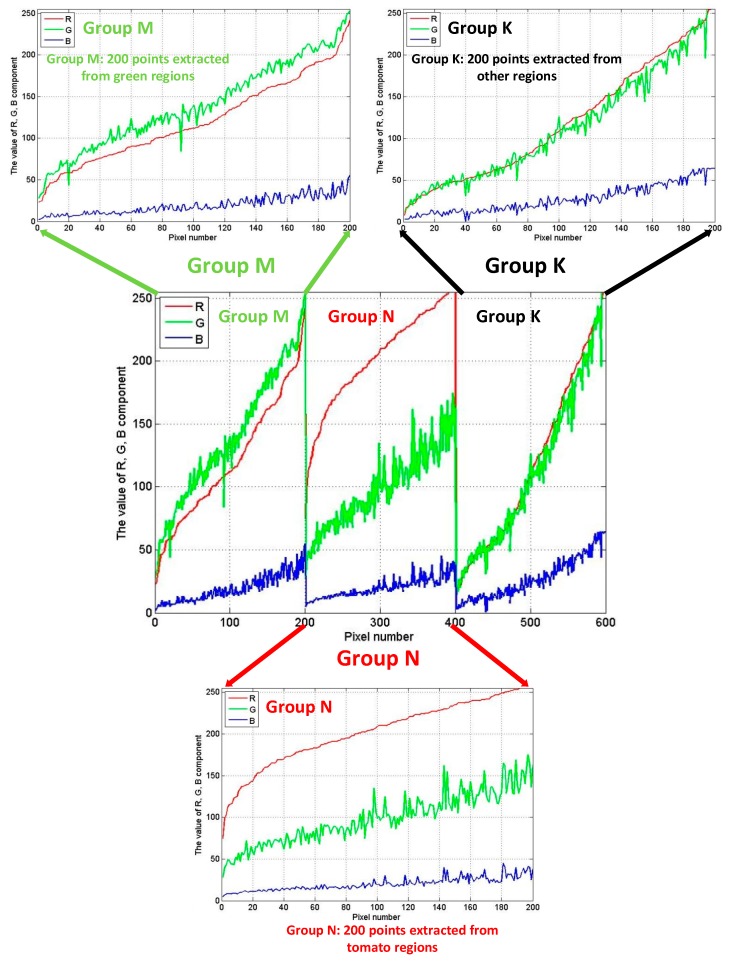
The R, G, B components of three groups M, N and K after reducing B’s proportion.

Then, we observed the values of pixel samples in groups M, N and K again after reducing the component B (shown in Figure 4). The difference between the components R and G can be seen visually. In group M, the component G is the main factor. While, the value of component R were greater than the value of component G in group N. Moreover, the values of components R and G are similar in group K. Depend on the difference above and the influence of component B, we set the parameters *α* to 1.00 and *γ* to 1.00. The value of Equation (1) is meaningful when component R occupies a large enough and decisive proportion (such as the samples in group N) and the result of Equation (1) is 0 in other cases (such as the samples in groups M and K). Above all, we set the three constant parameters *α*, *β*, *γ* to 1.00, 0.25, 1.00 in Equation (1).

**Figure 5:**
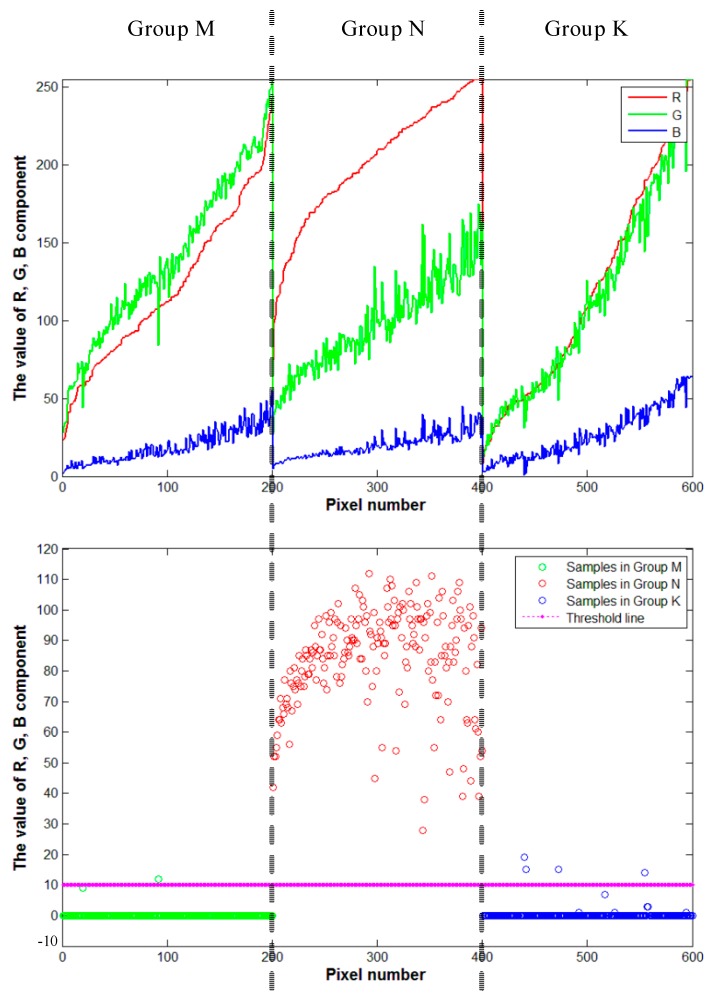
The results of Equation (1) for sample points.

Finally, we present the results of Equation (1) which the parameters had been determined on the coordinate axis (shown in Figure 5). It can be clearly seen that the most results of the samples in group N were above 20 value. Meanwhile, others were below 20 value. After discussion, we decided to loosen the threshold to 10 so that to extracted the target region entirely (even sacrifice a little accuracy in the first layer strategy). So, we set the threshold value to 10 as the separation parameter in Equation (2).

**Figure 6:**
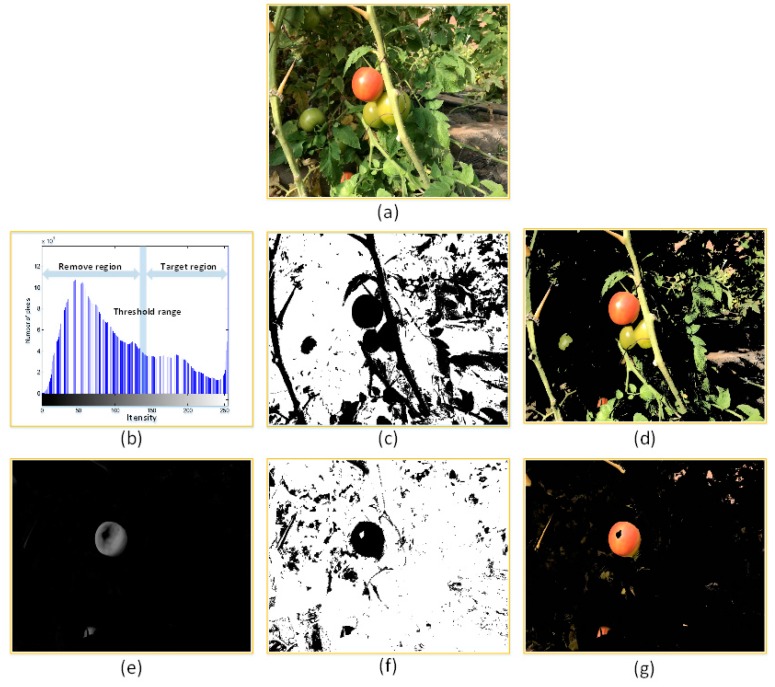
Results and comparison, for the cases in which only the R component was used and the integrated colour parameter. (**a**) Example image of ripening tomatoes. (**b**) Histogram of the image’s red component. (**c**) Segmentation mask image (Component R only). (**d**) Performance of the algorithm that uses only the R component. (**e**) Black-and-white image obtained using the integrated colour parameter method. (**f**) Segmentation mask image (Components R, G, B were used). (**g**) Result obtained using the integrated colour parameter method.

This strategy quickly and efficiently removed regions that exhibited significant differences in terms of their colour characteristics from target regions, based on the high colour contrast between tomato-containing and background regions. Using the conventional method, the R component is the main measure that determines the background removal. In the course of our studies, we found considering the R component only did not satisfy our requirement on the first layer strategy. Different environments, illumination intensity variations, and shadows all affect images. Figure 6b,c shows the corresponding red-channel histogram and the mask image segmentation result. The target region can be obtained using threshold processing by selecting a suitable threshold. However, some areas (e.g., some leaves and stems) with strong exposure satisfy the conditions that cannot be filtered out, which introduce many interference pixels into the mask image (as shown in Figure 6d).

By continuous improvement and experimentation, the integrated colour parameter which shows in Equation (1) calculated from the R, G, B components were used as the threshold of segmentation for background removal. It is feasible to use the integrated colour parameter to account for the three colour components. The R, G, B components account for different proportions in the target and non-target regions. In target regions, the R component plays a more significant role than the G and B components, while the opposite is true for non-target regions. Figure 6e,f shows the results when the integrated parameter was used for selecting target region and for creating the processing mask figure. It shows that the presence of interference pixels is significantly reduced compared with the result obtained when only the R component was used (as shown in Figure 6g).

### 3.2. Feature Weight Analysis and Determination

**Figure 7:**
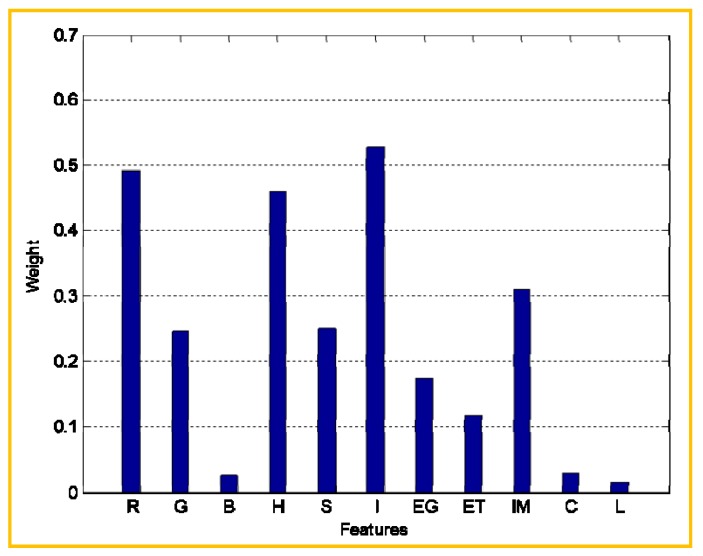
Resulting weight vector for the eleven extracted features. EG: Energy; ET: Entropy; IM: Inertial moment; C: Correlation; L: Local smoothing.

In the feature extraction step, relevant features were extracted from images in the training set, and eleven features were retained. Target regions (the areas that contains images of fruits) and non-target regions exhibit obvious differences, not only in terms of colour, but also in terms of textural features. In addition, different features contribute to different extents in the classification step. If these different contributions are not accounted for, or if all features have the same weight coefficients during the pattern recognition step, feature discrimination will not be possible. Therefore, the weight vector needs to account for the different contributions of different features to the classification process; this is likely to improve the recognition accuracy of test set images. Figure 7 shows the results of the feature weight analysis for the eleven analysed features (H, S, I, R, G, B, H, entropy, energy, correlation, inertial moment, and local smoothing). Figure 7 shows that not all of the extracted features contribute equally to the process of target region identification. Some features, such as R, H, I, and inertial moment, significantly contribute to the classification performance of the algorithm. On the other hand, B, correlation, and local smoothing features contribute less than other features. Additional insights can be obtained by analysing the weight vector diagram. For example, R component plays a much more important role than B and G components, which is easy to understand. However, it is difficult to directly determine the characteristics of intensity in an image with respect to texture. Clearly then, the weight analysis step is very apparent in the algorithm: its beneficial effect on the final results is obvious. In present work, the weights of the eleven analysed features are closely related to their significant status in the research. The feature weights were used in the classification step to build and train a weighted RVM classifier. The classification results suggest that our proposed method that combines colour features, textural features, and a weighted RVM classifier, can minimize the recognition error due to the existence of image regions with the same colour as our target regions.

In this study, we analyzed the multi-color features of the target region and combine texture features to form the feature vector. For color features data, we extracted color features (H, S, I components) from hue saturation value color space in order to avoid the influence of different light conditions. For texture features data, we extracted multi-modality texture features to distinguish the target region from the non-target region that have similar color features with target region. Above all, we use multi-color and multi-modality data feature vectors to overcome difficulties so that achieve the segmentation of target region.

### 3.3. Identification Results Using the Weighted RVM Classifier

To identify image regions that contain images for tomatoes, we used the weighted RVM classifier that was based on the features that were extracted from target regions in training set images; this analysis step constituted the second-layer identification strategy in the present work. This strategy was used for achieving recognition of tomatoes in images to combine with the approximate tomato-containing regions that were identified using the first-layer strategy, as well as for creating a mask figure for subsequent steps.

During the feature analysis, the weight vector that was obtained using the I-RELIEF algorithm on the eleven features was used for generating and training the weighted RVM classifier, along with the eleven features.

In the training step, the features were extracted from 9 × 9 image regions. The pixels of the relevant regions were assigned manually (the target regions were labelled as 0, the non-target regions including stem, calyx, and background were labelled as 1). Using the Bayes formula, the posterior probability distribution vector was generated. The features of the test set images are critically bound to the probability vector.

During the test step, systematic probabilistic results were obtained by calculating the vector that was obtained from the training step and the vectors of features that were obtained from regional blocks. The value of the latter can be taken to approximately indicate the classification success of pixel blocks (A pixel block represented by this group feature vector is non-target area when the value is under 50%, and a pixel block represented by this group feature vector is target area when the value is over 50%). Therefore, the probabilistic output of the weighted RVM classifier was used as a screening condition to scan and classify the feature vectors extracted by pixel blocks so as to obtain the mask diagram for the second-layer strategy, and the results would be shown in Section 3.4.

**Figure 8:**
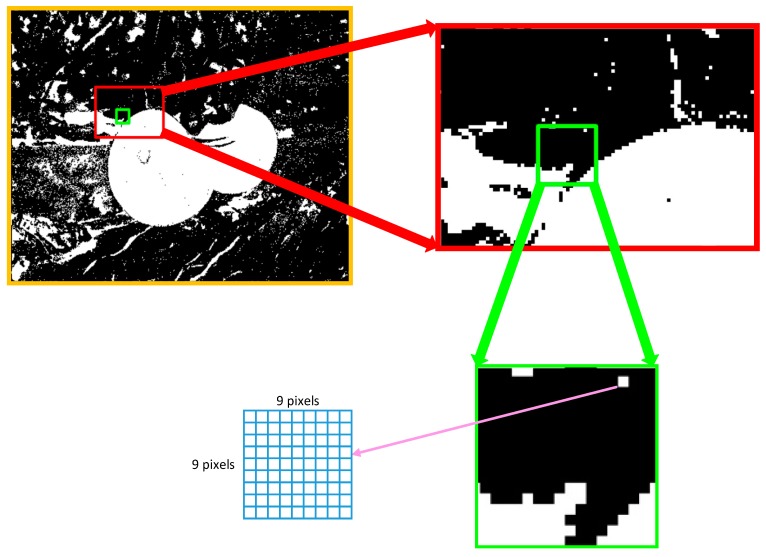
Demonstration of block segmentation for tomato recognition.

It is worth mentioning image regionalization. Owing to a large amount of experimental data, using 9 × 9 pixel blocks rather than single pixels not only simplifies the extraction of features, but also expedites the construction of the mask diagram. Using 9 × 9 pixels blocks has two significant advantages: (1) reduction of noise in feature vectors, and (2) significant reduction of computation time. This pixel block analysis was mainly used for extraction of features during the training and testing steps, and the test image scanning analysis was used during the testing step. In Figure 8**,** pixel blocks are clearly visible in the amplified view of the image.

The experimental results show that the background has similar colour characteristics as the region that contains the images of tomatoes region in the red-background image. Thus, our machine learning-based algorithm clearly overcomes same-colour problems that arise owing to the interference of colours. However, the performance is not ideal. Some weak light areas are difficult to be classified by RVM classifiers, because there is a lack of reflection in the areas of weak light that appear black and brown. After experimenting with training set characteristics, it was found this failed identification was not related to experimental error. In this study, images in the test set were analysed in terms of the characteristics extracted from the test set images, and the following preliminary conclusion was obtained: the above-mentioned errors in general appeared in non-target regions, for which feature extraction from test set images was incomplete. One or more features were assigned invalid values, which negatively affected the classifier’s performance on image recognition.

### 3.4. Results of the Bi-Layer Classification Strategy Algorithm and Final Results

**Figure 9:**
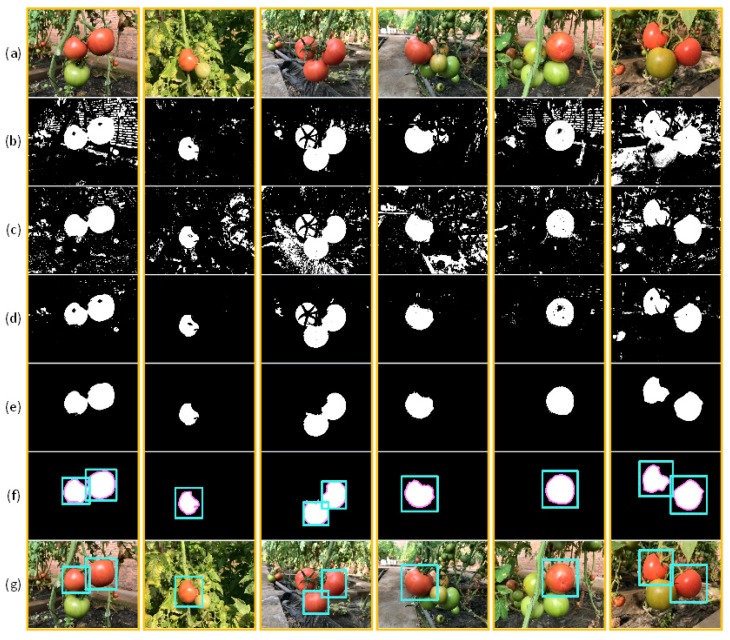
Process diagrams for sections in the algorithm. (**a**) Example images of ripening tomatoes. (**b**) Result of the first layer strategy based on the chromatic difference analysis. (**c**) Result of the second layer strategy based on the weighted RVM classifier. (**d**) Result of combining the two classification steps. (**e**,**f**) Results obtained after small areas were filled and contour detection. (**g**) Result obtained after marking the original image.

After completing the two-layer strategy, the mask diagrams formed by the two-layer strategy were combined. Figure 9b shows the resulting mask of the colour difference analysis. Figure 9c shows the results for the second identification using the weighted RVM classifier, while Figure 9d shows the fusion mask for the two layers strategies. The two mask diagrams affect each other and filter each other’s incorrectly recognized regions. The pixels that are identified by two strategies as non-target areas will be filled directly, then the small areas smaller than the average number of pixels in the mask image will be filled with morphological transform. The filling results are shown in Figure 9e. Use the location information obtained from the binary figure (e.g., Figure 9f), the corresponding position is marked in the original image, as shown in Figure 9g.

Figure 9g shows also that some obvious fruits were not recognized by the proposed algorithm. By performing root cause analysis of the image recognition process, we found that tomato areas were cut into several small areas in the small area filling process owing to the length of the blade, which resulted in several tomato-containing image regions being filled, which in turn precluded identification of some tomatoes. 

**Figure 10:**
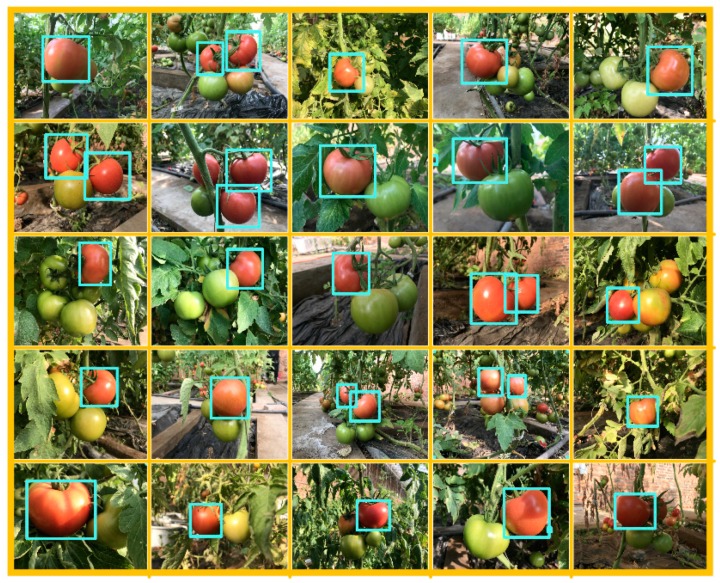
The testing results of the proposed algorithm.

Above all, the whole process of this algorithm was completed and the parts of finals results were shown in Figure 10. Through the first layer strategy, we can extract the approximate tomato regions by chromatic difference analysis. We realised goals of this strategy because component R occupies a large enough and decisive proportion during chromatic difference analysis. The negative influence of variation light will be reduced by subtraction when the lighting conditions change sharply for the target region. Nevertheless, the colours of non-target region may tend to be yellow or others colours and the value of component R may be enhanced, which result in error recognition in non-target region will be increased. We can still get the approximate tomato regions from numerous images acquired under intense light conditions by first layer strategy which support that the first layer using the chromatic difference analysis is feasible; For the second layer strategy, we adopted RVM classifier which more applicable and with less restrictions to recognize and segregate images. The feature sets include 11 dimensional features were composed of colour feature and textural feature. Multi-feature fusion enables classifier to fit more situations. Operation of pixels block speeds up the process of feature extraction and classification and reduces the error factors created form the single pixel. Weight vectors obtained from the weight analysis can present the contribution ratio of the 11 features. From the results, the recognitions of the target region in the second layer strategy are expected: It can separate the target region from images and eliminate interference factors such as stems or leaves regions. However, the phenomenon of false recognition was still existent in the regions where the features extraction was invalid or the range of features variation were less enough to be identified between target region and non-target region; Small area filling and edge detection were performed on the final mask images after the fi-layer and sec-layer strategies. It will make a good effect by using the number of regional edge points as filter objects to abstract the independent regions in mask image then filling them. It can eliminate the regions which conform to the requirement of features but do not conform to the distance in reality quickly and accurately. The problem found in this section is that target region may be divided into several independent regions by leaves or stems, and then be filled. Although this is rare, we cannot ignore the existence of such phenomenon. However, such problem will reduce or even disappear with the change of shooting angle, so we think it will be improved with the movement of harvesting robots in practical application.

Although every section mentioned above may cause false recognition, the algorithm can recognize the tomato regions from images with an accuracy rate of 94.90%. Such accuracy rate may not be particularly outstanding, but from the process in our algorithm, the overall segmentation results are acceptable and reasonable for we took many factors into consideration and avoided many misunderstandings. In the future, the summary and analysis of each section can help us to improve the accuracy of identification.

## 4. Conclusions

A tomato recognition algorithm that employs a two-layer classification strategy based on the fusion of features and a weighted related vector machine classifier has been proposed for tomato detection.

The strategy in the first part of our analysis amounted to comparing the results of the distribution analysis of a single R component and the colour difference analysis results. The colour difference analysis information parameter was used for preliminary screening of images and for constructing the result mask of the first layer strategy.

In the second strategy, the images were again analysed using the weighted RVM classifier and drawing the mask images. In this step, we first used the iterative RELIEF algorithm to analyse the relevant image characteristics and generate the weighted vector of the characteristics according to the contributions of different characteristics. When training the classifier, the weighted vector that affected the formation of the kernel function matrix was used to validate the image recognition using the feature vector obtained on the training set using the Bayes formalism.

The small area that below the edge points mean is filled with the morphological transform to obtain the result mask image. Next, edge detection was used to determine the target areas in the final mask diagram, and these locations were mapped back onto the corresponding positions in the original images to achieve target area masks.

The results of the first layer classification strategy show that the segmentation of comprehensive colour parameters by chromatic difference analysis can be achieved but that instances of misidentification still exist, especially so for regions with similar colour characteristics. The results of the second layer classification strategy show that using the weighted RVM classifier can eliminate interference between similar colour regions, and recognition rate can be improved by using texture-related features. However, the performance on areas for which some features attained invalid values was bad. Therefore, a recognition algorithm combined with the two-layer classification strategy yields fast and accurate recognition of tomatoes. 

The analysis of more than 100 experimental images shows the feasibility of the proposed tomato recognition method. The success rate of the proposed method on the tomato recognition task was 94.90%, and the identification process took 2.94 s on the average, which is suitable for implementation of robotic tomato harvesting. The recognition time of the current algorithm is still quite long, mostly owing to the vast amount of experimental data. In actual production, the volume of data acquired using digital cameras will need to be reduced, according to the actual implementation, to make the method cost-efficient. In the present work, the identification time was deemed to be sufficiently short for actual applications. Future work will focus on fault identification during the second classification step, as well as on increasing the efficiency of the proposed algorithm.
